# *Pseudomonas aeruginosa *bloodstream infections: risk factors and treatment outcome related to expression of the PER-1 extended-spectrum beta-lactamase

**DOI:** 10.1186/1471-2334-6-52

**Published:** 2006-03-16

**Authors:** Andrea Endimiani, Francesco Luzzaro, Beatrice Pini, Gianfranco Amicosante, Gian Maria Rossolini, Antonio Q Toniolo

**Affiliations:** 1 Laboratorio di Microbiologia, Università dell'Insubria and Ospedale di Circolo, I-21100 Varese; 2 Dipartimento di Scienze e Tecnologie Biomediche, Università di L'Aquila, I-67100 L'Aquila; 3 Dipartimento di Biologia Molecolare, Sezione di Microbiologia, Università di Siena, I-53100 Siena, Italy

## Abstract

**Background:**

Bloodstream infection (BSI) due to Pseudomonas aeruginosa (Pa) has relevant clinical impact especially in relation to drug resistance determinants. The PER-1 extended-spectrum beta-lactamase (ESBL) is a common enzyme conferring high-level resistance to anti-pseudomonal cephalosporins. Risk factors and treatment outcome of BSI episodes caused by PER-1-positive Pa (PER-1-Pa) strains were compared to those caused by ESBL-negative Pa isolates (ESBL-N-Pa).

**Methods:**

Twenty-six BSI cases due to ceftazidime-resistant Pa strains have been investigated. MIC values of anti-pseudomonal drugs were determined by the Etest method (AB Biodisk, Solna, Sweden). The double-disk synergy test was used to detect ESBL production. PCR amplification and DNA sequencing were used to characterize ESBL types. Clinical records of BSI-patients were examined retrospectively. Demographic data, underlying diseases (McCabe-Jackson classification and Charlson weighted index), risk factors, antimicrobial therapy, and treatment outcome were evaluated in cases due to ESBL-positive and cases due to ESBL-N-Pa isolates. Unpaired Student's t-test, Mann-Whitney U-test, Fisher's exact test and the χ^2 ^test were used for statistical analysis.

**Results:**

Nine Pa isolates expressed the PER-1 ESBL; the remaining 17 isolates did not produce ESBLs. Severe sepsis (*P *= 0.03), bladder and intravascular catheters (both *P *= 0.01), immunosuppressive therapy (*P *= 0.04), and mechanical ventilation (*P *= 0.03) were significantly associated with BSI due to PER-1-Pa. Empirical treatment (*P *= 0.02) and treatment after ID/AST (*P *< 0.01) were rarely adequate in PER-1-Pa cases. With regard to treatment outcome, 77.8% BSI cases due to PER-1-Pa *vs*. 28.6% cases due to ESBL-N-Pa isolates failed to respond (*P *< 0.03). All cases due to PER-1-Pa that were treated with carbapenems (alone or in combination with amikacin) failed to respond. In contrast, 7/8 cases due to ESBL-N-Pa given carbapenems were responders.

**Conclusion:**

Therapeutic failure and increased hospital costs are associated with BSI episodes caused by PER-1-Pa strains. Thus, recognition and prompt reporting of ESBL-production appears a critical factor for the management of patients with serious *P. aeruginosa *infections.

## Background

*Pseudomonas aeruginosa *(Pa) is commonly responsible for nosocomial infections, including surgical site infection, urinary tract infection, pneumonia, and bloodstream infection (BSI) [1,2]. In particular, this organism is responsible for 3–7% BSI cases [1,3-5]. Notably, high morbidity and mortality rates (range 27% to 48%) have been observed in critically ill patients [5,6].

Resistance to multiple drugs is a common feature of hospital-acquired Pa strains [4,7,8]. Low permeability of the outer membrane proteins (OMPs), production of the inducible AmpC chromosomal β-lactamase, and multi-drug efflux systems contribute to the intrinsic resistance of this species [7]. Thus, drugs suitable against Pa infections are limited to aminoglycosides (e.g., gentamicin, amikacin), fluoroquinolones (ciprofloxacin remains the most active), selected β-lactams (e.g., ceftazidime, carbapenems), and one β-lactam/β-lactamase inhibitor combination (piperacillin/tazobactam) [4,5,7,8]. Unfortunately, acquired resistance to different categories of the anti-pseudomonal agents is also possible and has been widely illustrated [7]. In particular, resistance to β-lactams is very common and is due to mutations amplifying intrinsic resistance mechanisms (i.e., AmpC), and/or acquisition of additional β-lactamase genes by horizontal transfer [7,8].

Acquired β-lactamases found in Pa isolates can be classified into three different groups: i) narrow-spectrum enzymes (e.g., PSE-1/4) that efficiently degrade penicillins and cefoperazone; ii) extended-spectrum β-lactamases (ESBL) (e.g., PER-1, VEB-1, GES-1/2) that also degrade cephems and monobactams; iii) metallo-β-lactamases (MBL) (e.g., IMP-, VIM-type) that efficiently degrade all anti-pseudomonal β-lactams with the exception of monobactams [7,8].

The PER-1 ESBL is a class A enzyme conferring high-level of resistance to anti-pseudomonal β-lactams and is one of the most frequently ESBL detected in Pa [9-11]. PER-1 was first detected in a urinary Pa isolate in a Turkish patient in 1991 [9,12]. Subsequently, it was frequently recognized in Pa and *Acinetobacter *spp. isolates [13,14]. In 1997, our group was the first to detect PER-1-positive Pa (PER-1-Pa) isolates outside the geographical area of origin [15]. To date, the PER-1 ESBL has been reported also from other areas of Europe and Asia [16-19], and in different genera and species (i.e., *Alcaligenes faecalis*, *Salmonella tiphymurium*, *P. mirabilis*, *Providencia *spp.) [20-24].

Risk factors, empirical treatment and treatment outcome of BSI due to Pa have been investigated by different authors [25-28]. Particular emphasis has been posed on clinical differences observed between multi-drug resistant (MDR) and susceptible Pa isolates [6]. In contrast, the clinical features of BSI caused by ESBL-positive Pa strains have not been investigated.

We had the opportunity of studying 26 cases of BSI caused by ceftazidime-resistant Pa (CAZ-R-Pa) of which 9 were caused by PER-1-Pa strains. Microbiological and clinical data have been compared to those of 17 BSI cases due to ESBL-negative Pa isolates (ESBL-N-Pa). The impact of PER-1 expression on risk factors and treatment outcome was evaluated.

## Methods

### Sample collection and clinical data

Blood cultures were processed at the Microbiology Laboratory of the Ospedale di Circolo e Fondazione Macchi (Varese, Italy), an 800-bed university hospital that provides care in specialized areas such as heart surgery, neurosurgery, hemato-oncology, kidney transplantation, intensive care units (ICU). The study was performed in conformity with the World Medical Association Declaration of Helsinki and with the approval of the Hospital Ethics Committee.

Clinical records of patients who developed BSI due to CAZ-R-Pa strains in the period 1998–2004 were examined retrospectively. The following data were studied: demographic data, primary source of infection leading to secondary BSI (when confirmed by culture), antimicrobial agents administered during hospitalization, cause of death. To categorize the severity of underlying diseases, McCabe & Jackson classification scheme and Charlson weighted index were used [29,30]. Severity of septicemia was classified as described [31].

The following predisposing conditions (when present for at least 72 hours before BSI onset) were also investigated: mechanical ventilation, intravascular and bladder catheters, drainages (i.e., thoracic or abdominal), esophago-gastroscopy, bronchoscopy, parenteral feeding, angiography, renal dialysis, nephrostomy, and neutropenia. In addition, previous surgery, previous use of antibiotics, use of immunosuppressive drugs (e.g., corticosteroids, antineoplastic agents) were taken into account when administered for at least two weeks before BSI onset.

### Definitions

Primary BSI refers to bacteremia for which no source of infection was documented. BSI was defined as secondary when laboratory evidence showed infection by the same organism at a distant site. Antibiotic treatment was defined as empirical when given before species identification (ID) and antimicrobial susceptibility tests (AST). Treatment was considered adequate when the responsible Pa strain was subsequently found susceptible to the administered drug(s).

Treatment outcome of patients was classified as follows: (i) "complete response", resolution of fever, leukocytosis, and local signs and symptoms of infection; (ii) "partial response", improvement of fever, leukocytosis, and local signs and symptoms of infection without complete resolution; (iii) "relapse", recurrence of infection with the same organism at any body site within 1 month after discontinuation of therapy; (iv) "treatment failure", absence of resolution or worsening of signs and symptoms of infection; (v) "not assessable", incomplete records or death of the patient within 72 hours of BSI onset. BSI-patients who had complete or partial response to treatment were considered as "responders", whereas those who had relapse or treatment failure were considered as "nonresponders". Treatment outcome was attributed to the drug(s) that were administered after receiving the microbiological report containing ID and AST results. Death was considered as attributable to BSI when occurred within 7 days of BSI diagnosis or when the patient was still under treatment. The 28-day mortality rate was also reported. Follow-up of patients that were discharged or transferred to a different hospital within 1 month of BSI onset was performed in collaboration with the caring physicians.

### Microbiological methods

Blood cultures were incubated for at least 5 days using the BACTEC 9240 instrument (Becton Dickinson Diagnostic Systems, Sparks, MD). Results were interpreted according to guidelines of the Centers for Diseases Control and Prevention [32]. Isolates obtained from blood cultures of the same patient after at least 7 days of a previous BSI episode were considered as causing a new BSI episode [32].

ID and AST were routinely achieved by the Sceptor System (Becton Dickinson) during 1998–2002 or the Phoenix System (Becton Dickinson) since 2003. ID results were confirmed using the ID32GN strips (Bio-Mérieux, Marcy L'Étoile, France). Pa isolates causing BSI were stored at -80°C in Todd-Hewitt broth supplemented with 20% glycerol for further investigations.

Minimum inhibitory concentration (MIC) values were determined by the Etest method (AB Biodisk, Solna, Sweden). Susceptibility categories were determined according to current criteria of the Clinical Laboratory Standards Institute, CLSI [33]. The reference *P. aeruginosa *strain ATCC 27853 was used as control.

### Screening of ESBL-producing isolates

To screen for ESBL producing strains, CAZ-R-Pa isolates (MIC > 8 μg/ml) were tested using the double-disk synergy test on Mueller-Hinton agar plates with disks containing 30 μg of aztreonam, ceftazidime, and cefepime placed 20 mm apart (center to center) around a disk containing amoxicillin (20 μg) plus clavulanic acid (10 μg). Enhancement of the inhibition zone – indicating synergy between clavulanic acid and any test drugs – was taken as presumptive evidence of ESBL production. To detect possible MBL producers, CAZ-R-Pa were also screened with Etest strip containing imipenem and imipenem plus EDTA (AB Biodisk).

### β-lactamase characterization by isoelectric focusing (IEF)

Supernatants of crude bacterial lysates obtained by sonication were studied with IEF using a polyacrylamide gel containing Pharmalyte anpholines (pH range, 3.5 to 10.0) (Pharmacia, Milan, Italy). Gels were run for 3 hours at 10 W and 4°C using a Multiphor II system (Pharmacia). β-lactamase bands were evidenced with 1 mM nitrocefin as chromogenic substrate (Oxoid, Milan, Italy) in 0,1 M phosphate buffer (pH 7.0) [15]. Estimations of pI values were made by comparison with extracts of reference bacterial strains producing the PER-1 (5.3), TEM-1 (5.4), TEM-2 (5.6), TEM-3 (6.3), and SHV-1 (7.6) enzymes.

### Molecular studies for confirming the PER-1 determinant

Colony blot hybridization and Southern blot hybridization were carried out as described [15]. The probe used in filter hybridization experiments was a PCR-generated amplicon comprising the entire *bla*_PER-1 _open reading frame labeled with ^32^P by the random priming technique [12,20].

PCR amplification of *bla*_PER _alleles was performed as described on crude DNA extracts [20]. The *bla*_PER-1 _and *bla*_PER-2 _genes were distinguished by digestion with *Pvu*II and *Stu*I; the amplicon sequence was determined on crude amplification products as described [20].

### Statistical analysis

Statistical analysis was performed using the Statistica PC software (StatSoft, Tulsa, OK). Variance by logistic regression was calculated in order to compare patient groups infected by PER-1-Pa or ESBL-N-Pa isolates. The Student's unpaired t-test was used to compare continuous variables, and the Mann-Whitney U-test to compare not normally distributed continuous variables. Fisher's exact test and the χ^2 ^test were used to compare treatment outcome between patient groups. Differences were considered statistically significant when 2-tailed *P *value was ≤ 0.05.

## Results

### Microbiological data of isolates causing BSI

During the study period (January 1998 to September 2004), 122 strains of Pa causing BSI in adult patients were stored at -80°C. On the basis of Etest method, CAZ-R-Pa isolates were 26/122 (21.3%). Suspect MBL-producing strains were not detected. The double-disk synergy test showed that 9/26 isolates (34.6%) were ESBL-positive.

IEF analysis showed that supernatants of the 26 CAZ-R-Pa isolates exhibited a band at pI 8.0–8.4 associated to the expression of the chromosomal AmpC enzyme. All double-disk positive strains (9/26) showed an additional band at pI 5.3 compatible with the expression of the PER-1 enzyme. Molecular analysis confirmed that all isolates showing nitrocephin hydrolysis at pI 5.3 produced the PER-1 ESBL (data not shown).

As shown in Table 1, PER-1-Pa isolates were consistently resistant to anti-pseudomonal drugs: ceftazidime (MIC >256 μg/mL), cefepime (MIC >48 μg/mL), carbapenems (MIC >8 μg/mL), piperacillin/tazobactam (MIC >256 μg/mL), ciprofloxacin (MIC >32 μg/mL), and gentamicin (MIC >256 μg/mL). Only 4 isolates had amikacin MIC values into the susceptibility range (MIC, 16 μg/mL). ESBL-N-Pa strains showed reduced susceptibility to all extended-spectrum cephalosporins; carbapenems were the most active β-lactams (70.6% susceptible isolates). Concerning other drugs, resistance to ciprofloxacin and gentamicin was common (52.9 and 76.5%, respectively). Amikacin-resistant strains were only 3/17 (17.6%).

**Table 1:** Antimicrobial susceptibility test and molecular results for all ceftazidime-resistant *P. aeruginosa *isolates (n = 26) responsible of bloodstream infections (BSI)

Isolates	MIC of agents (μg/ml) ^a^										Type of enzymes produced
		
	PIP	TZP	CAZ	FEP	ATM	IMP	MEM	CIP	AMK	GEN	
338/98	> 256	> 256	> 256	128	> 256	8	32	> 32	64	> 256	AmpC, PER-1
LS034/99	> 256	> 256	> 256	> 256	> 256	> 32	> 32	> 32	64	> 256	AmpC, PER-1
140/99	> 256	> 256	> 256	64	> 256	16	32	> 32	64	> 256	AmpC, PER-1
47/00	> 256	> 256	> 256	128	> 256	16	8	> 32	16	> 256	AmpC, PER-1
324/00	> 256	> 256	> 256	> 256	> 256	> 32	16	> 32	16	> 256	AmpC, PER-1
A360/00	> 256	> 256	> 256	> 256	> 256	> 32	16	> 32	16	> 256	AmpC, PER-1
527/00	> 256	> 256	> 256	> 256	> 256	> 32	32	> 32	16	> 256	AmpC, PER-1
2126/01	> 256	> 256	> 256	64	> 256	16	8	> 32	32	> 256	AmpC, PER-1
A150/02	> 256	> 256	> 256	48	> 256	> 32	32	> 32	> 256	> 256	AmpC, PER-1
299/98	> 256	128	32	64	32	2	2	0.094	4	8	AmpC
77/99	> 256	64	64	16	64	4	2	0.5	8	8	AmpC
251/00	> 256	128	16	32	16	2	4	> 32	128	> 256	AmpC
746/00	> 256	64	16	64	16	8	8	> 32	96	> 256	AmpC
2497/01	> 256	128	16	128	16	2	1	0.125	8	> 256	AmpC
2544/01	256	64	32	128	64	16	2	0.5	16	> 256	AmpC
A372/02	64	32	16	32	32	0.5	0.5	> 32	32	> 256	AmpC
A480/02	> 256	64	64	16	8	> 32	16	0.094	1	4	AmpC
659/02	96	32	32	16	16	1	0.5	> 32	8	> 256	AmpC
694/02	128	64	16	8	16	4	8	> 32	16	> 256	AmpC
A276/03^b^	64	32	32	16	16	1	0.5	> 32	16	> 256	AmpC
A562/03^b^	64	16	32	16	16	1	0.5	> 32	16	> 256	AmpC
A173/03^b^	32	4	32	16	16	1	0.5	> 32	4	8	AmpC
A189/03 ^c^	32	16	32	16	8	1	0.5	> 32	8	> 256	AmpC
A590/03	> 256	128	64	8	64	32	8	0.125	0.5	2	AmpC
A424/04	> 256	64	64	4	32	4	2	0.094	1	4	AmpC
A567/04 ^c^	128	64	64	16	16	1	0.5	0.125	0.5	2	AmpC

^a ^MIC values were obtained using the Etest method (AB Biodisk). Abbreviations of antimicrobial agents and breakpoints for susceptibility (S) and resistance (R) are given: PIP, piperacillin (S ≤ 64, R ≥ 128); TZP, piperacillin plus tazobactam (S ≤ 64, R ≥ 128); CAZ, ceftazidime (S ≤ 8, R ≥ 32); FEP, cefepime (S ≤ 8, R ≥ 32); ATM, aztreonam (S ≤ 8, R ≥ 32); IPM, imipenem (S ≤ 4, R ≥ 16); MEM, meropenem (S ≤ 4, R ≥ 16); CIP, ciprofloxacin (S ≤ 1, R ≥ 4); AMK, amikacin (S ≤ 16, R ≥ 64); GEN, gentamicin (S ≤ 4, R ≥ 16).

^b ^These strains caused three different BSI in the same patient.

^c ^Only microbiological data were available. Therefore, these 2 cases were excluded from statistical analysis of demographic data, risk factors and treatment outcome.

### Risk factors for BSI caused by PER-1-Pa isolates

Twenty-six cases of BSI due to CAZ-R-Pa isolates were observed in 24 patients (one patient was affected by 3 different episodes of bacteremia). Since clinical data of 2 patients affected by one BSI episode were not available (cases A189/03 and A567/03), statistical analysis was performed considering only 22 patients affected by 24 BSI cases.

Demographic data and severity of underlying diseases of BSI-patients infected by PER-1-Pa and ESBL-N-Pa isolates are shown in Table 2. Chance of developing hospital-acquired BSI and greater mean length of hospital stay (MLHS) might be related to infection by PER-1-positive strains, though differences were not statistically significant (*P *= 0.07 and *P *= 0.09, respectively). Clinical parameters of BSI cases due to PER-1-Pa and ESBL-N-Pa isolates are shown in Table 3. Severe sepsis (*P *= 0.03), bladder and intravascular catheters (both, *P *= 0.01), immunosuppressive therapy (*P *= 0.04), and mechanical ventilation (*P *= 0.03) were significantly associated with BSI due to PER-1-Pa isolates. Empirical treatment and treatment after ID/AST were significantly less adequate in the PER-1-Pa than in the ESBL-N-Pa group (*P *= 0.02 and *P *<0.01, respectively).

**Table 2:** Demographic data and severity of the underlying disease of analyzed patients (n = 22) with BSI due to ceftazidime-resistant *P. aeruginosa *strains: difference between PER-1-positive (PER-1-P-Pa) and ESBL-negative Pa (ESBL-N-Pa) isolates.^a^

Demographic and clinical parameters	PER-1-Pa	ESBL-N-Pa	*P *value ^b^
No. BSI-patients	9	13	-
Male/Female	7/2	11/2	-
Age, mean year ± SD	58.0 ± 22.1	63.4 ± 17.7	NS
Patient location			
- Medical	3 (33.3)	7 (53.8)	NS
- Surgical	0 (0.0)	2 (15.4)	NS
- ICU	6 (66.7)	4 (30.8)	0.10
McCabe & Jackson groups			
- Nonfatal	6 (66.7)	5 (38.5)	NS
- Ultimately fatal	1 (11.1)	5 (38.5)	NS
- Rapidly fatal	2 (22.2)	3 (23.1)	NS
Charlson weighted index, mean ± SD	2.9 ± 2.57	3.5 ± 2.6	NS
Previous hospitalizations (during the last 12 months)	5 (55.6)	9 (69.2)	NS
Previous use of antibiotics (during the last 12 months)	5 (55.6)	7 (53.8)	NS
Hospital-acquired BSI	9 (100)	9 (69.2)	0.07
Mean length of hospital stay (MLHS), mean ± SD (days)	69.3 ± 58.0	41.6 ± 35.3	0.09
- MLHS before BSI diagnosis, mean ± SD (days)	22.9 ± 8.0	17.1 ± 24.0	NS
- MLHS after the BSI, mean ± SD (days)	46.4 ± 57.5	24.4 ± 20.6	0.11
28-day mortality	2 (22.2)	1 (7.7)	NS
Mortality attributable to BSI (within 7 days of onset)	1 (11.1)	0 (0.0)	NS

^a ^Data are expressed as no. (%) of patients, unless otherwise indicated.

^b ^-, not calculated; NS, not significant.

**Table 3:** Clinical parameters of investigated BSI cases (n = 24) due to ceftazidime-resistant *P. aeruginosa *strains: difference between PER-1-positive (PER-1-Pa) and ESBL-negative (ESBL-N-Pa) isolates.^a^

Clinical parameters	PER-1-Pa	ESBL-N-Pa	*P *value ^b^
No. BSI episodes	9	15^c^	-
Severity of septicemia			
- Sepsis	5 (55.6)	12 (80.0)	NS
- Severe sepsis	4 (44.4)	1 (6.7)	0.03
- Septic shock	0 (0.0)	2 (13.3)	NS
Empirical treatment provided	8 (88.9)	14 (93.3)	NS
- Adequate	1 (11.1)	9 (60.0)	0.02
Adequate treatment after ID / AST ^d ^results	1 (11.1)	12 (80.0)	< 0.01
Predisposing factors for BSI infection			
- Bladder catheter	9 (100)	7 (46.7)	0.01
- Previous use of antibiotics	8 (88.9)	9 (60.0)	NS
- Intravascular catheter	9 (100)	7 (46.7)	0.01
- Immunosuppressive therapy	5 (55.6)	5 (33.3)	0.04
- Previous surgery	7 (77.8)	5 (33.3)	NS
- Drainages	2 (22.2)	5 (33.3)	NS
- Mechanical ventilation	6 (66.7)	3 (20.0)	0.03
- Esophagogastroscopy	1 (11.1)	2 (13.3)	NS
- Other	4 (44.4) ^e^	4 (26.6) ^f^	-
Overall secondary BSI	5 (55.6)	10 (66.7)	NS
- Urinary tract	2 (22.2)	3 (20.0)	NS
- Respiratory tract	3 (33.3)	4 (26.7)	NS
- IV catheter	1 (11.1)	1 (6.7)	NS
- Wounds	0 (0.0)	4 (26.7)	NS

^a ^Data are expressed as no. (%) of patients.

^b ^-, not calculated; NS, not significant.

^c ^One patient was affected by three different BSI episodes.

^d ^ID, identification; AST, antimicrobial susceptibility tests.

^e ^Bronchoscopy (n = 1), dialysis (n = 1), parenteral feeding (n = 1), angiografhy (n = 1), and neutropenia (n = 1).

^f ^Bronchoscopy (n = 1), dialysis (n = 1), parenteral feeding (n = 1), nephrostomy (n = 1), and neutropenia (n = 1).

### Treatment outcome of BSI caused by PER-1-Pa isolates

Clinical parameters, antimicrobial regimens and treatment outcome for each BSI episode due to PER-1-Pa and ESBL-N-Pa isolates are summarized in Table 4 and Table 5, respectively.

**Table 4:** Clinical parameters, antimicrobial regimens and treatment outcome of patients with bloodstream infection (BSI) due to PER-1-positive *P. aeruginosa *(PER-1-Pa) isolates

Isolate	Age (yr)	Sex ^a^	McCabe & Jackson ^b^	Charlson weighted index	Primary source(s) of infection ^c^	Severity of septicemia	Empirical treatment		Treatment administered after ID and AST ^d ^results			Treatment outcome ^e^
									
							Agent (daily dose)	Adequate	Agent (daily dose)	Timing from BSI onset	Duration (days)	
338/98	58	M	NF	1	Unknown	Severe sepsis	SXT (10 ml × 3)	No	AMK (0.5 g × 2) FEP (2 g × 3)	Day 4 Day 7	10 11	FA
LS034/99	60	M	NF	0	LRTI	Severe sepsis	IPM (1.5 g × 3)	No	IPM (1.5 g × 3) AMK (0.5 g × 2)	Day -21 Day -2	53 8	RE
140/99	69	M	NF	1	Unknown	Severe sepsis	CTX (2 g × 3) IPM (0.5 g × 3)	No	Not changed	Day -15 Day 0	20 5	FA
47/00	19	F	NF	6	LRTI, IVC	Sepsis	MEM (1 g × 2) AMK (1 g)	Yes	MEM (1 g × 3) AMK (1 g)	Day 1	6 18	FA
324/00	27	M	NF	0	Unknown	Sepsis	CIP (0.4 g × 2)	No	Not changed	Day 0	13	RE
A360/00	72	M	RF	6	LRTI	Sepsis	None	No	MEM (0.5 g × 4) AMK (0.75 g)	Day 3 Day 3	13 18	RE
527/00	60	M	NF	3	Unknown	Sepsis	IPM (0.5 g × 4)	No	Not changed	Day -9	13	CR
2126/01	67	F	RF	6	UTI	Sepsis	CRO (2 g)	No	IPM (1 g × 3)	Day 3	8	PR
A150/02	90	M	UF	3	UTI	Severe sepsis	CIP (0.2 g × 2) TZP (2 g × 2)	No	IPM (0.5 g × 2)	Day 1	11	RE

Abbreviations for antimicrobial agents: SXT, trimethoprim-sulfametoxazole; PIP, piperacillin; TZP, piperacillin plus tazobactam; CRO, ceftriaxone; FEP, cefepime; IPM, imipenem; MEM, meropenem; CIP, ciprofloxacin; AMK, amikacin; GEN, gentamicin.

^a ^Sex: F, female; M, male.

^b ^McCabe & Jackson classification: NF, nonfatal; UF, ultimately-fatal; RF, rapidly-fatal.

^c ^Primary source(s) of infection: LRTI, lower respiratory tract infection; IVC, intravascular catheter colonization; UTI, urinary tract infection.

^d ^ID, identification; AST, antimicrobial susceptibility test.

^e ^Treatment outcome: CR, complete response; PR, partial response; FA, failure; RE, relapse.

**Table 5:** Clinical parameters, antimicrobial regimens and treatment outcome of patients with bloodstream infection (BSI) due to ESBL-negative *P. aeruginosa *(ESBL-N-Pa) isolates

Isolate	Age (yr)	Sex ^a^	McCabe & Jackson ^b^	Charlson weighted index	Primary source(s) of infection ^c^	Severity of septicemia	Empirical treatment		Treatment administered after ID and AST ^d ^results			Treatment outcome ^e^
									
							Agent (daily dose)	Adequate	Agent (daily dose)	Timing from BSI onset	Duration (days)	
299/98	40	M	NF	0	LRTI, WI	Septic shock	IPM (1 g × 3)	Yes	Not changed	Day 1	12	FA
77/99	76	M	UF	4	LRTI, UTI	Sepsis	IPM (0.5 × 3)	Yes	Not changed	Day 0	14	CR
251/00	55	M	UF	6	Unknown	Sepsis	IPM (0.5 g × 2)	Yes	Not changed	Day -1	1	NA
746/00	64	M	NF	2	UTI	Sepsis	PIP (2 g × 2) CIP (0.5 g)	No	IPM (0.5 g × 2)	Day 5	7	CR
2497/01	34	M	NF	1	Unknown	Sepsis	PIP (2 g × 2)	No	CIP (0.5 g × 2)	Day 5	3	CR
2544/01	79	F	UF	3	Unknown	Sepsis	NET (0.15 g × 2)	No	IPM (0.5 g × 3)	Day 1	13	CR
A372/02	67	M	RF	7	WI	Severe sepsis	CIP (0.5 g × 2) AMK (0.5 g × 2)	No	IPM (0.5 × 2)	Day 4	9	CR
A480/02	69	F	NF	0	LRTI, WI	Sepsis	CIP (0.4 g × 2)	Yes	Not changed	Day 1	10	FA
659/02	71	M	RF	8	UTI	Sepsis	IPM (0.5 g × 2)	Yes	Not changed	Day 0	8	CR
694/02	93	M	UF	3	IVC	Sepsis	PTZ (2.25 g × 3)	Yes	PTZ (2.25 g × 3) AMK (0.5 g × 2)	Day 0 Day 6	7 2	CR
A276/03^f^	76	M	NF	3	Unknown	Sepsis	None	No	IPM (1 g × 3)	Day 3	10	PR
A562/03^f^	76	M	NF	3	Unknown	Sepsis	PTZ (2.25 g × 3)	Yes	Not changed	Day 0	15	CR
A173/03^f^	76	M	NF	3	Unknown	Sepsis	PTZ (2.25 g × 3)	Yes	Not changed	Day -1	29	CR
A590/03	64	M	RF	6	LRTI, WI	Sepsis	IPM (1 g × 3) AMK (0.75 g)	Yes	AMK (1 g)	Day 2	8	RE
A424/04	36	M	UF	3	Unknown	Septic shock	IPM (0.5 g × 4)	No	MEM (1 g × 2)	Day 4	19	FA

Abbreviations for antimicrobial agents: NET, netilmicin; PIP, piperacillin; TZP, piperacillin plus tazobactam; CRO, ceftriaxone; FEP, cefepime; IPM, imipenem; MEM, meropenem; CIP, ciprofloxacin; AMK, amikacin; GEN, gentamicin.

^a ^Sex: F, female; M, male.

^b ^McCabe & Jackson classification: NF, nonfatal; UF, ultimately-fatal; RF, rapidly-fatal.

^c ^Primary source(s) of infection: LRTI, lower respiratory tract infection; WI, wound infection; UTI, urinary tract infection; IVC, intravascular catheter colonization.

^d ^ID, identification; AST, antimicrobial susceptibility test.

^e ^Treatment outcome: CR, complete response; PR, partial response; FA, failure; RE, relapse; NA, not assessable.

^f ^These strains caused three different BSI in the same patient.

Patients infected by PER-1-Pa strains were treated as follows: carbapenems (n = 4), carbapenems plus amikacin (n = 3), cefepime plus amikacin (n = 1), and ciprofloxacin (n = 1). Those infected by ESBL-N-Pa strains were treated with: carbapenems (n = 9), ciprofloxacin (n = 2), piperacillin/tazobactam (n = 2), piperacillin/tazobactam plus amikacin (n = 1), and amikacin (n = 1). One patient given imipenem was excluded from statistical analysis since he was transferred to a different hospital just one day after BSI onset (case #251/00).

Overall, 7/9 (77.8%) cases due to PER-1-Pa vs. 4/14 (28.6%) cases due to ESBL-N-Pa isolates failed to respond (P < 0.03). In particular, 5/7 BSI cases due to PER-1-Pa strains failed to respond to carbapenems (alone or in combination with other drugs), while 7/8 cases due to ESBL-N-Pa isolates responded satisfactorily to carbapenems. With regard to amikacin, 4/4 BSI cases due to PER-1-Pa vs. 1/2 cases due to ESBL-N-Pa strains failed to respond. Notably, 3/3 cases due to PER-1-Pa that were treated with carbapenems plus amikacin failed to respond.

## Discussion

The clinical impact of ESBL-production in *P. aeruginosa *isolates causing BSI received little attention since Pa produces a small percentage of BSI cases and this species rarely harbors ESBLs [1,3-5]. The increasing circulation of ESBL determinants in this species underlines the need of studying this issue [19,14,24]. In some geographic areas such as Northern Italy, high incidence of PER-1-positive *P. aeruginosa *has been recently reported [19]. This retrospective analysis was undertaken to evaluate the clinical features of BSI episodes due to PER-1-Pa isolates. Twenty-six BSI episodes due to CAZ-R-Pa isolates (nine of which produced the PER-1 enzyme) that occurred over a 7-year period have been studied. Five out of 9 PER-1-Pa strains were clonally related and were collected during an outbreak that occurred at our hospital [15].

As reported in the case of ESBL-producing enterobacteria [24], risk factors such as bladder and intravascular catheters, immunosuppressive therapy, mechanical ventilation were significantly associated with BSI episodes caused by PER-1-Pa isolates. This result may be explained by the fact that cases due to PER-1-Pa were most prevalent among ICU patients. The overall MLHS and MLHS after BSI onset were greater in cases due to PER-1-Pa than in cases due to ESBL-N-Pa isolates (in both cases the MLHS was increased by approximately 20 days). This finding highlights the impact of ESBL-positive Pa strains on hospital costs [34]. In Italy, the average hospital day cost is approximately 500 Euro (Agenzia per i Servizi Sanitari Regionali, ). Thus, the hospital cost of each patient with PER-1-Pa-induced BSI is approximately 10,000 Euro higher than that of patients with BSI due to ESBL-negative Pa.

The PER-1-Pa isolates collected at our institution were resistant to virtually all anti-pseudomonal drugs. This finding justifies the observed high rates of inadequate empirical and rational treatments. Inadequate treatment was, in fact, significantly higher in cases due to PER-1-Pa as compared to cases due to ESBL-N-Pa. It should be emphasized that inappropriate initial therapy in Pa BSI has been associated with worse outcome [27,28]. Our data, however, do not confirm the above findings. In fact, despite that only 10/24 (41.7%) cases infected by CAZ-R-Pa isolates received adequate empirical treatment, the overall mortality rate was 13.6% (cases #140/99, #2126/01, and #A372/02). In particular, 8/9 patients infected with PER-1-Pa isolates received inadequate empirical treatment, but only 1 (case #140/99) died for causes attributable to BSI. In this group, however, most patients who survived were young and had non-fatal underlying diseases [27].

With regard to PER-1 expression, significant responsibility of this determinant on mortality has been reported from Turkey [35]. The present results fail to confirm this conclusion. Not significantly different death rates were in fact observed between patients infected with PER-1-Pa and ESBL-N-Pa isolates. Though this may be related to the low number of BSI cases investigated by us, the Turkish study investigated infections of different body sites comparing those caused by PER-1-positive isolates with those produced by Pa strains with variable resistance profiles [35]. Our study was instead focused only on BSI cases due to CAZ-R-Pa isolates harboring or not the PER-1 determinant.

The overall treatment outcome of BSI-patients infected by ESBL-negative strains was significantly better than that of patients infected by PER-1-Pa (*P *< 0.03). In particular, monotherapy with carbapenems was adequate only in BSI cases due to ESBL-N-Pa isolates. Concerning amikacin, only 4/9 isolates of PER-1-Pa were in the susceptibility range, but the two BSI-patients receiving adequate treatment with this drug (cases A360/00 and 47/00) were classified as nonresponders. In the latter two cases, amikacin MIC (16 μg/mL) was close to the susceptibility breakpoint (≤ 16 μg/mL) [33]. Treatment failure could be ascribed to the inability of the drug to reach adequate tissue concentrations, especially at sites where high numbers of bacteria were present.

Though combination therapy is usually recommended for severe Pa infections [2,7,27], three patients affected by BSI due to PER-1-Pa isolates failed to respond to carbapenems plus amikacin. Treatment outcome results appear to indicate that combinations of carbapenems plus aminoglycosides are not reliable options for severe infections caused by PER-1-Pa isolates. Mimoz and colleagues studying experimental pneumonia in rats showed that imipenem plus amikacin produced optimal results against a PER-1-Pa isolate [36]. The observed inability of imipenem plus amikacin to resolve BSI cases might reflect the expression of additional resistance mechanisms. It is hardly believable that the association of AmpC and PER-1 enzymes is responsible for carbapenem resistance in *P. aeruginosa*. Additional mechanisms (OMPs alterations, efflux pumps) might thus be operative in carbapenem-resistant strains. A role of OMPs modifications appear likely, since 6/9 PER-1-Pa isolates showed imipenem MIC values higher than those of meropenem. This may indicate that mutations leading to decreased expression of the OprD porin could be responsible for differences in susceptibility to imipenem and meropenem [7].

## Conclusion

Our study shows that therapeutic failure and increased hospital costs are associated with BSI episodes due to PER-1-Pa strains. The existing therapeutic options for Pa isolates carrying ESBL determinants are still inadequate. Thus, recognition and prompt reporting of ESBL-production appears a critical factor for the management of patients with serious *P. aeruginosa *infections. The possible role of old antimicrobials such as colistin should be re-evaluated [37].

## Competing interests

The author(s) declare that they have no competing interests.

## Authors' contributions

AE analyzed the clinical data and prepared the manuscript together with FL.

BP performed microbiological phenotypic tests.

GA carried out molecular assays.

GMR and AQT coordinated the study and helped writing the manuscript.

All authors read and approved the final version of the manuscript.

## Pre-publication history

The pre-publication history for this paper can be accessed here:



## References

[B1] Gaynes R, Edwards JR, National Nosocomial Infections Surveillance System (2005). Overview of nosocomial infections caused by Gram-negative bacilli. Clin Infect Dis.

[B2] Pollack M, Mandell GL, Bennett JE, Dolin R (2000). *Pseudomonas aeruginosa*. Principles and practice of infectious diseases.

[B3] Luzzaro F, Vigano EF, Fossati D, Grossi A, Sala A, Sturla C, Sbudelli M, Toniolo A, AMCLI Lombardia Hospital Infections Study Group (2002). Prevalence and drug susceptibility of pathogens causing bloodstream infections in Northern Italy: a two-year study in 16 hospitals. Eur J Clin Microbiol Dis.

[B4] Biedenbach DJ, Moet GJ, Jones RN (2004). Occurrence and antimicrobial resistance pattern among bloodstream infection isolates from the SENTRY Antimicrobial Surveillance Program (1997–2002). Diagn Microbiol Infect Dis.

[B5] Wisplinghoff H, Bischoff T, Tallent SM, Seifert H, Wenzel RP, Edmond MB (2004). Nosocomial bloodstream infections in US hospitals: analysis of 24,179 cases from a prospective nationwide surveillance study. Clin Infect Dis.

[B6] Kang CI, Kim SH, Park WB, Lee KD, Kim HB, Kim EC, Oh MD, Choe KW (2005). Risk factors for antimicrobial resistance and influence of resistance on mortality in patients with bloodstream infection caused by *Pseudomonas aeruginosa*. Microb Drug Res.

[B7] Rossolini GM, Mantegoli E (2005). Treatment and control of severe infections caused by multiresistant *Pseudomonas aeruginosa*. Clin Microbiol Infect.

[B8] Navon-Venezia S, Ben-Ami R, Carmeli Y (2005). Update on *Pseudomonas aeruginosa *and *Acinetobacter baumannii *infections in the heathcare setting. Curr Opin Infect Dis.

[B9] Nordmann P, Ronco E, Naas T, Duport C, Michel-Briand Y, Labia R (1993). Characterization of a novel β-lactamase from *Pseudomonas aeruginosa*. Antimicrob Agents Chemother.

[B10] Vahaboglu H, Saribas S, Akbal H, Ozturk R, Yucel A (1998). Activities of cefepime and five other antibiotics against nosocomial PER-1-type and/or OXA-10-type β-lactamase-producing *Pseudomonas aeruginosa *and *Acinetobacter *spp. J Antimicrob Chemother.

[B11] Weldhagen GF, Poirel L, Nordmann P (2003). Ambler class A extended-spectrum β-lactamases in *Pseudomonas aeruginosa *: novel developments and clinical impact. Antimicrob Agents Chemother.

[B12] Nordmann P, Naas T (1994). Sequence analysis of PER-1 extended-spectrum β-lactamase from *Pseudomonas aeruginosa *and comparison with class A β-lactamases. Antimicrob Agents Chemother.

[B13] Vahaboglu H, Öztürk R, Aygün G, Coskunkan F, Yaman A, Kaygusuz A, Leblebicioglu H, Balik I, Aydin K, Otkun M (1997). Widespread detection of PER-1-type extended-spectrum β-lactamases among nosocomial *Acinetobacter *and *Pseudomonas aeruginosa *isolates in Turkey: a nationwide multicenter study. Antimicrob Agents Chemother.

[B14] Kolayli F, Gacar G, Karadenizli A, Sanic A, Vahaboglu H, The study Group (2005). PER-1 is still widespread in Turkish hospitals among *Pseudomonas aeruginosa *and *Acinetobacter *spp. FEMS Microbiol Lett.

[B15] Luzzaro F, Mantegoli E, Perilli M, Lombardi G, Orlandi V, Orsetti A, Amicosante G, Rossolini GM, Toniolo A (2001). Dynamics of a nosocomial outbreak of multidrug-resistant *Pseudomonas aeruginosa *producing the PER-1 extended-spectrum β-lactamase. J Clin Microbiol.

[B16] De Champs C, Poirel L, Bonnet R, Sirot D, Chanal C, Sirot J, Nordmann P (2002). Prospective survey of beta-lactamases produced by ceftazidime-resistant *Pseudomonas aeruginosa *isolated in a French hospital 2000. Antimicrob Agents Chemother.

[B17] Claeys G, Verschraegen G, De Baere T, Vaneechoutte M (2000). PER-1 β-lactamase producing *Pseudomonas aeruginosa *in an intensive care unit. J Antimicrob Chemother.

[B18] Yong D, Shin JH, Kim S, Lim Y, Jum JH, Lee K, Chong Y, Bauernfeind A (2003). High prevalence of PER-1 extended-spectrum β-lactamase-producing *Acinetobacter *spp. in Korea. Antimicrob Agents Chemother.

[B19] Pagani L, Mantegoli E, Migliavacca R, Nucleo E, Pollini S, Spalla M, Daturi R, Romero E, Rossolini GM (2004). Multifocal detection of multidrug-resistant *Pseudomonas aeruginosa *producing the PER-1 extended-spectrum β-lactamase in northtern Italy. J Clin Microbiol.

[B20] Pereira M, Perilli M, Mantegoli E, Luzzaro F, Toniolo A, Rossolini GM, Amicosante G (2000). PER-1 extended-spectrum β-lactamase production in an *Alcaligenes faecalis *clinical isolate resistant to expanded-spectrum cephalosporins and monobactams from a hospital in northern Italy. Microb Drug Resist.

[B21] Vahaboglu H, Hall LMC, Mulazimoglu L, Dodanli S, Yildirim I, Livermore DM (1995). Resistance to extended-spectrum cephalosporins caused by PER-1 β-lactamase, in *Salmonella tiphymurium *from Istambul, Turkey. J Med Microbiol.

[B22] Pagani L, Migliavacca R, Pallecchi L, Matti C, Giacobone E, Amicosante G, Romero E, Rossolini GM (2002). Emerging extended-spectrum beta-lactamases in *Proteus mirabilis*. J Clin Microbiol.

[B23] Bahar G, Erac B, Mert A, Gulay Z (2004). PER-1 production in a urinary isolate of *Providencia rettgeri*. J Chemother.

[B24] Paterson DL, Bonomo RA (2005). Extended-spectrum β-lactamases: a clinical update. Clin Microbiol Rev.

[B25] Kang CI, Kim SH, Kim HB, Park SW, Choe YJ, Oh MD, Kim EC, Choe KW (2003). *Pseudomonas aeruginosa *bacteremia: risk factors for mortality and influence of delayed receipt of effective antimicrobial therapy on clinical outcome. Clin Infect Dis.

[B26] Kang CI, Kim SH, Park SW, Lee KD, Kim HB, Kim EC, Oh MD, Choe KW (2005). Clinical features and outcome of patients with community-acquired *Pseudomonas aeruginosa *bacteremia. Clin Microbiol Infect.

[B27] Chamot EC, Boffi El Amari E, Rohner P, Van Delden C (2003). Effectiveness of combination antimicrobial therapy for *Pseudomonas aeruginosa *bacteremia. Antimicrob Agents Chemother.

[B28] Micek ST, Lloyd AE, Ritchie DJ, Reichley RM, Fraser VJ, Kollef MH (2005). *Pseudomonas aeruginosa *bloodstream infection: importance of appropriate initial antimicrobial treatment. Antimicrob Agents Chemother.

[B29] McCabe WR, Jackson GG (1962). 1. Etiology and ecology. Arch Intern Med.

[B30] Charlson ME, Pompei P, Ales KL, MacKenzie CR (1987). A new method of classifying prognostic comorbidity in longitudinal studies: development and validation. J Chron Dis.

[B31] American College of Chest Physicians/Society of Critical Care Medicine Consensus Conference (1992). Definitions of sepsis, organ failure and guidelines for the use of innovative therapies in sepsis. Crit Care Med.

[B32] Garner JS, Jarvis WR, Emori TG, Horan TC, Hughes JM (1988). CDC definitions for nosocomial infections. Am J Infect Control.

[B33] Clinical and Laboratory Standard Methods (2005). Performance standards for antimicrobial susceptibility testing: fifteenth informational supplement.

[B34] Carmeli Y, Troillet N, Karchmer AW, Samore MH (1999). Health and economic outcomes of antibiotic resistance in *Pseudomonas aeruginosa*. Arch Intern Med.

[B35] Vahaboglu H, Coskunkan F, Tansel O, Ozturk R, Sahin N, Koksal I, Kocazeybek B, Tatman-Otkun M, Leblebicioglu H, Ozinel MA, Akalin H, Kocagoz S, Korten V (2001). Clinical importance of extended-spectrum β-lactamase (PER-1-type)-producing *Acinetobacter *spp. and *Pseudomonas aeruginosa *strains. J Med Microbiol.

[B36] Mimoz O, Elhelali N, Lèotard S, Jacolot A, Laurent F, Samii K, Petitjean O, Nordmann P (1999). Treatment of experimental pneumonia in rats caused by a PER-1 extended-spectrum β-lactamase-producing strain of *Pseudomonas aeruginosa*. J Antimicrob Chemother.

[B37] Li J, Nation RL, Milne RW, Turnidge JD, Coulthard K (2005). Evaluation of colistin as an agent against multi-resistant Gram-negative bacteria. Int J Antimicrob Agents.

